# Should unaffected female *BRCA2* pathogenic variant carriers be told there is little or no advantage from risk reducing mastectomy?

**DOI:** 10.1007/s10689-019-00142-8

**Published:** 2019-08-23

**Authors:** D. Gareth Evans, Sacha J. Howell, Anthony Howell

**Affiliations:** 1 NW Genomic Laboratory hub, Manchester Centre for Genomic Medicine, Manchester University Hospitals NHS Foundation Trust, Manchester, M13 9WL UK; 2 Division of Evolution and Genomic Sciences, School of Biological Sciences, Faculty of Biology, Medicine and Health, University of Manchester, Manchester Academic Health Science Centre, Manchester, UK; 3 Prevent Breast Cancer Centre, Wythenshawe Hospital Manchester, University NHS Foundation Trust, Wythenshawe, Manchester, M23 9LT UK; 4 Manchester Breast Centre, The Christie NHS Foundation Trust, Wilmslow Road, Manchester, M20 4BX UK; 5 Division of Cancer Sciences, Faculty of Biology, Medicine and Health, University of Manchester, Manchester Academic Health Science Centre, Manchester, UK

A recent article from the Dutch HEBON study showing a small but significant survival advantage for *BRCA1*, but a non-significant one for *BRCA2* pathogenic variant carriers undergoing bilateral risk reducing mastectomy (BRRM) has sparked a large amount of debate [1]. The conclusion of the article states ‘for *BRCA2* mutation carriers BC (breast cancer) surveillance may be as effective as BRRM regarding breast cancer-specific survival. Although the number of events are small especially for the analyses on breast cancer-specific mortality our findings may support a more individualized counseling based on BRCA mutation type regarding the difficult choice between BRRM and BC surveillance.’ This prompted a fair degree of media response including a Dutch newspaper article in *Algemeen Dagblad* on 29/07/2019 with the headline "Preventive breast surgery appears not to be necessary". The original press release that prompted this headline appears on the lead author’s website (https://www.erasmusmc.nl/nl-nl/kankerinstituut/patientenzorg/artikelen/preventieve-borstamputatie-verbetert-overleving-bij-brca1). The link carries an apparent quote from the lead author stating that ‘For the *BRCA2* mutation carriers struggling with the choice between intensive control and preventive removal, it can be a relief to know that with regard to the chance of dying from breast cancer, intensive control appears to be just as effective as breast removal’. The newspaper article prompted a response from the Dutch Breast Cancer Association https://borstkanker.nl/nl/nieuws/vrouwen-met-brca-schrikken-van-artikel-ad and the Netherlands Association for Clinical Genetics (VKGN) (http://www.vkgn.org/nieuws/onderzoek-naar-preventieve-borstamputatie). Both responses made it clear that the option of BRRM is not only driven by an as yet marginal potential reduction in the risk of dying from breast cancer but also to avoid a cancer diagnosis and the resultant treatment.

In the original article the overall survival (ORs) for *BRCA1* and *BRCA2* are not that different, at 0.40 and 0.45 respectively, so the claims are overstated that there is no apparent benefit for *BRCA2*. There was one breast cancer death in *BRCA1* carriers undergoing BRRM compared to 20 undergoing surveillance, with no deaths in *BRCA2* carriers undergoing BRRM with seven in those having surveillance. The breast cancer specific mortality rate for *BRCA1* was significantly reduced at 0.2 (0.02–1.1) compared to 1.7 (1.1–2.6) for surveillance (HR 0.06 (95% CI 0.01–0.46) with a lower rate for *BRCA2* in the surveillance group of 0.9 (0.4–1.9), but unmeasurable in the BRRM group due to zero BC deaths. It is clear from the data available that *BRCA2* is not as powered as *BRCA1* and therefore the ORs are not (yet) significant. Follow up time post BRRM in women years is, by our calculations based on median follow up, less than half in *BRCA2* carriers at around 2920 years compared to 6209 years in *BRCA1* carriers. Similarly the observation period in those undergoing surveillance was longer in *BRCA1* carriers at around 9207 years compared to 6355 years for *BRCA2*. It was not possible to find data on length of follow up post breast cancer diagnosis, but given the relatively short median follow ups of 9.3 years for *BRCA1* and 8.6 years for *BRCA2* and that cancers may have occurred late in follow up, the mean follow up time is likely only around 5 years. Whilst this follow up for the majority of *BRCA1* carriers with grade 3 triple negative cancers (TNC) (77% TNC, 74% grade 3) may be sufficient to determine long term survival, since most of the deaths with this tumour type occur within 5 years of diagnosis, this is not the case for *BRCA2* (78% ER+, 63% Grade 1 or 2). Oestrogen receptor positive cancers continue to relapse at about 1% per year until at least 20 years. This is shown by our own data (Table 1) in Manchester where over 50% of breast cancer related deaths occurred after 5 years follow up and nearly 29% after 10 years. This would suggest that the seven breast cancer deaths in the *BRCA2* surveillance group is likely to more than double. Although Kaplan-Meier analysis was relatively reassuring with 98% breast cancer related survival to age 65 years in the *BRCA2* surveillance group this does not take into account the extra *BRCA2* breast cancer related deaths that will occur, nor that more *BRCA2* carriers in the surveillance group will develop breast cancer. Realistically if followed for 40 years from 25–65 years of age at least 50% of *BRCA2* carriers will develop breast cancer with many developing contralateral disease if not undertaking BRRM at diagnosis or soon after. In the study 144/739 (19%) developed breast cancer in the *BRCA2* surveillance group over a median of only 8.6 years [1]. A more realistic and still optimistic estimate of the breast cancer specific survival in a *BRCA2* surveillance group by age 65 years would be 95%.

**Table 1:** Breast cancer deaths in women with *BRCA1* or *BRCA2* pathogenic variants in women diagnosed since 1990 in Manchester.

	Breast cancer related deaths	All deaths in women with breast cancer including non-cancer	BC deaths < 5 years	% of all BC deaths	BC deaths > 10 years (%)	
*BRCA1*	195	265	118	60.51	31	15.90
*BRCA2*	237	278	106	44.73	68	28.69

Although there is support for the authors claims of better *BRCA2* projected deaths from breast cancer from simulated models [2, 3] and from small studies of actual survival [4], an earlier report from some members of the authorship showed no survival advantage of MRI screening over controls in *BRCA2* [5].

The authors’ overview of the limitations of their study, concentrate mainly on why the 0.45 survival benefit may overestimate any mortality risk reduction. These include that BRRM may have been carried out on ‘healthier’ women, and that screening may not have been very well adhered to given the 41% combined interval and symptomatic proportions in *BRCA2*. No limitations are described in terms of length of follow up from breast cancer, and indeed, the authors state that the strengths include the sufficient numbers of *BRCA1* and *BRCA2* mutation carriers ‘with long enough follow-up’. We disagree with this assessment for *BRCA2*, although we do agree that the benefits of BRRM may be more marginal in *BRCA2* carriers taking into account the lower penetrance and better stage distribution seen in *BRCA2* versus *BRCA1*. However, as pointed out by the Dutch Breast Cancer Association and from two of the authors of the study [1] representing the Netherlands Association for Clinical Genetics (VKGN), the media have completely ignored the driver of women’s feelings that they are ‘sitting on a ticking time bomb’ and the strong wish to avoid a breast cancer diagnosis and treatment. It is a concern that the media in search of good ‘headlines’ often use press releases without contacting authors before publishing ‘sensational’ headlines. That said neither the press release nor the original article [1], emphasise the other reasons for BRRM. There is therefore a need for better communication between scientific article authors and the media before news articles with misleading headlines are published that can seriously upset patients who have undertaken procedures such as BRRM.

There may be many drivers for the decision to undergo BRRM. Women may choose this option because of family experience, particularly related to breast cancer diagnosis [6, 7], but also from recent false positive screens with biopsy showing no cancer [7]. Age is also a strong factor with younger women more likely to choose the option [6–8], which may make sense in terms of risk as they have more of their risk to live through. Uptake of BRRM is not always immediate with more than 50% of uptake occurring after 2 years from genetic testing [7]. There may be cultural reasons for differences in uptake as reflected in international variations [6, 8, 9] showing higher uptake in North America, the Netherlands, the UK and most of Northern Europe with low uptake in France the Mediterranean, Eastern Europe and Israel. This may not only reflect the attitudes of women, but those of their clinicians [10]. Clearly there may be a difference in uptake if clinical staff discussing BRRM are not keen on the intervention and may make only cursory reference to it as an option or dismiss it, if mentioning it at all. On the other hand some may be overenthusiastic and this may lead to potentially ‘inappropriate’ increased uptake. Trends may also have changed over time [6, 8] reflecting better access to MRI screening and other preventive options, as well as to better quality BRRM with immediate reconstruction. However, MRI leads to a higher false positive rate that is a known trigger for BRRM [7] and can be claustrophobic for some women reducing adherence. Decision aids to help women make choices regarding BRRM and surveillance are highly important (in part to offset the variation in counselling) [3], but these may need updating to reflect the potentially higher mortality in *BRCA1* carriers as pointed out in the HEBON study and by others [1, 11].

In addition to the wishes of many women to avoid a breast cancer diagnosis, the longer term costs of screening with MRI annually and of treating resultant cancers have also been overlooked as they are likely to substantially exceed those of BRRM. This is particularly the case with systemic therapies such as PARP inhibitors that have recently gained approval in advanced breast cancer and, if adjuvant trials are positive, will substantially increase the cost of therapy in all *BRCA* PV carriers, although they may also improve survival [12]. In summary, BRRM remains a valuable option for all women carrying *BRCA1* and *BRCA2* pathogenic variants. The information from the article provide additional data to aid decision making but caution must be taken in their interpretation.

## References

[1] Heemskerk-Gerritsen Bernadette A. M., Jager Agnes, Koppert Linetta B., Obdeijn A. Inge-Marie, Collée Margriet, Meijers-Heijboer Hanne E. J., Jenner Denise J., Oldenburg Hester S. A., van Engelen Klaartje, de Vries Jakob, van Asperen Christi J., Devilee Peter, Blok Marinus J., Kets C. Marleen, Ausems Margreet G. E. M., Seynaeve Caroline, Rookus Matti A., Hooning Maartje J. (2019). Survival after bilateral risk-reducing mastectomy in healthy BRCA1 and BRCA2 mutation carriers. Breast Cancer Research and Treatment.

[2] Kurian AW, Sigal BM, Plevritis SK (2010). Survival analysis of cancer risk reduction. strategies for BRCA1/2 mutation carriers. J Clin Oncol.

[3] Kurian AW, Munoz DF, Rust P, Schackmann EA, Smith M, Clarke L, Mills MA, Plevritis SK (2012). Online tool to guide decisions for BRCA1/2 mutation carriers. J Clin Oncol.

[4] Evans DG, Harkness EF, Howell A, Wilson M, Hurley E, Holmen MM, Tharmaratnam KU, Hagen AI, Lim Y, Maxwell AJ, Moller P (2016). Intensive breast screening in BRCA2 mutation carriers is associated with reduced breast cancer specific and all-cause mortality. Hered Cancer Clin Pract.

[5] Saadatmand S, Obdeijn IM, Rutgers EJ, Oosterwijk JC, Tollenaar RA, Woldringh GH (2015). Survival benefit in women with BRCA1 mutation or familial risk in the MRI screening study (MRISC). Int J Cancer.

[6] Metcalfe KA, Birenbaum-Carmeli D, Lubinski J, Gronwald J, Lynch H, Moller P, Ghadirian P, Foulkes WD, Klijn J, Friedman E, Kim-Sing C, Ainsworth P, Rosen B, Domchek S, Wagner T, Tung N, Manoukian S, Couch F, Sun P, Narod SA, Hereditary Breast Cancer Clinical Study Group (2008). International variation in rates of uptake of preventive options in BRCA1 and BRCA2 mutation carriers. Int J Cancer.

[7] Evans DG, Lalloo F, Ashcroft L, Shenton A, Clancy T, Baildam AD, Brain A, Hopwood P, Howell A (2009). Uptake of risk reducing surgery in unaffected women at high risk of breast and ovarian cancer is risk, age and time dependent. Cancer Epid Biomark Prev.

[8] Metcalfe K, Eisen A, Senter L, Armel S, Bordeleau L, Meschino WS, Pal T, Lynch HT, Tung NM, Kwong A, Ainsworth P, Karlan B, Moller P, Eng C, Weitzel JN, Sun P, Lubinski J, Narod SA, Hereditary Breast Cancer Clinical Study Group (2019). International trends in the uptake of cancer risk reduction strategies in women with a BRCA1 or BRCA2 mutation. Br J Cancer.

[9] Julian-Reynier C, Bouchard L, Evans G, Eisinger F, Foulkes W, Kerr B, Blanquaert I, Moatti J-P, Sobol H (2001). Women's Attitudes toward preventive strategies for Hereditary Breast/Ovarian Cancer risk differ from one country to another: differences between Manchester (UK), Marseilles (F) and Montreal (Ca). Cancer.

[10] Den Heijer M, van Asperen CJ, Harris H, Nippert I, Schmidtke J, Bouhnik AD, Julian-Reynier C, Evans DG, Tibben A (2013). International variation in physicians' attitudes towards prophylactic mastectomy—comparison between France, Germany, the Netherlands and the United Kingdom. Eur J Cancer.

[11] Evans DG, Howell A (2012). Are we ready for online tools in decision making for BRCA1/2 mutation carriers?. J Clin Oncol.

[12] Tung NM, Garber JE (2018). BRCA1/2 testing: therapeutic implications for breast cancer management. Br J Cancer.

